# The effect of high lateral position on antibiotic exposure duration in patients with severe traumatic brain injury: a retrospective observational cohort study

**DOI:** 10.3389/fmed.2025.1665953

**Published:** 2026-01-05

**Authors:** Zhongbao Lin, Dandan Chen, Mei Ye, Xincai Wang, Liman Qiu, Long Huang

**Affiliations:** 1 Department of Critical Care Medicine, Fuzhou University Affiliated Provincial Hospital, Fujian Provincial Hospital, Shengli Clinical Medical College of Fujian Medical University, Fuzhou, China; 2 Department of Respiratory and Critical Care Medicine, Fuzhou University Affiliated Provincial Hospital, Fujian Provincial Hospital, Shengli Clinical Medical College of Fujian Medical University, Fuzhou, China; 3 The United Innovation of Mengchao Hepatobiliary Technology Key Laboratory of Fujian Province, Mengchao Hepatobiliary Hospital of Fujian Medical University, Fuzhou, China

**Keywords:** antibiotic exposure, aspiration pneumonia, high lateral position, neurocritical care, severe traumatic brain injury

## Abstract

**Background:**

Patients with severe traumatic brain injury (sTBI) often require mechanical ventilation and have a high incidence of aspiration pneumonia, leading to prolonged antibiotic exposure. This study assesses the association between high lateral position (HLP) and antibiotic exposure duration in sTBI patients, and reports on safety and feasibility.

**Methods:**

This study retrospectively collected data from 138 patients with severe traumatic brain injury complicated by aspiration pneumonia who were treated in the intensive care unit from January 2023 to June 2024. Patients were divided into two groups based on whether they received high lateral position (HLP) therapy: the HLP group (*n* = 45) and the non-HLP group (*n* = 93). Univariate and multivariate linear regression analyses were used to identify independent risk factors associated with the duration of antibiotic use, and the association between HLP therapy and antibiotic use duration was evaluated by comparing the two groups.

**Results:**

Patients who received HLP had a shorter duration of antibiotic use; both univariate and multivariate regression analyses suggested an association between HLP and shorter duration of use. Univariate linear regression analysis showed that HLP, chronic obstructive pulmonary disease (COPD), arterial oxygen partial pressure, and oxygenation index were all associated with the duration of antibiotic use. Multivariate linear regression analysis further confirmed that HLP was independently associated with a shorter duration of antibiotic use (*β* = −2.58; 95% CI, −4.44 to −0.71; *p* = 0.008), while COPD was associated with a longer duration of use (β = 8.78; 95% CI, 4.42 to 13.13; *p* < 0.001).

**Conclusion:**

In this retrospective cohort, HLP was associated with a shorter duration of antibiotic exposure and showed trends toward fewer days of mechanical ventilation and a lower tracheostomy rate. Given the nonrandomized design and potential residual confounding, these findings are exploratory and should be confirmed in prospective randomized studies.

## Introduction

Severe traumatic brain injury (sTBI) affects more than 50 million individuals worldwide each year and is a leading cause of death and long-term disability, with substantial socioeconomic burden (1, 2). Patients with sTBI commonly experience impaired cough, secretion retention, and a heightened risk of aspiration due to neurogenic respiratory dysregulation (3, 4). Secondary pulmonary infection increases mortality three- to five-fold, underscoring the importance of early, targeted respiratory care (5). Optimizing respiratory management is therefore central to improving outcomes in sTBI, and enhanced airway care has been associated with shorter antibiotic courses, reduced multidrug-resistant (MDR) infections, and lower rates of failed extubation (6, 7).

In neurocritical care, secretion retention and ventilation–perfusion mismatch are major drivers of prolonged antibiotic therapy and pulmonary complications. Among non-pharmacological strategies, body positioning can be used to leverage gravity for mucus clearance and to improve V/Q matching (8). Prone positioning (PP) enhances oxygenation and aids secretion clearance in acute respiratory distress syndrome (9, 10), but its use in patients with acute brain injury is limited by concerns about intracranial pressure (ICP) and practical challenges. Several studies report moderate but significant ICP elevations during PP with premature termination in a proportion of patients (11), whereas others suggest acceptable changes in ICP (12). Proposed mechanisms include increased intra-abdominal pressure and restricted jugular or vertebral venous outflow, which may impair cerebral venous drainage (13). PP can also complicate bedside care (e.g., pressure injury risk, constraints on pupillary assessment) (14–16), further limiting its adoption in this population.

Given these limitations, high lateral positioning (HLP) is considered a more feasible alternative that may be friendlier to cerebral venous outflow. Executed at roughly 90° lateral tilt with 15–30° head-of-bed elevation and a neutral neck, HLP avoids abdominal compression and may help preserve ICP and cerebral perfusion pressure (CPP) stability while gravity supports secretion drainage—a mechanistic hypothesis that requires clinical testing. Preliminary evidence in neurosurgical cohorts suggests that, under appropriate angles with head elevation, lateral positioning does not significantly alter ICP or CPP (17), and the physiologic rationale that ~30° head elevation lowers ICP and supports CPP is relatively consistent (18). Hemodynamic modeling and observational signals further indicate that the lateral decubitus position may reduce extracranial venous resistance and optimize cerebral venous outflow (19). Although oxygenation benefits comparable to PP have been reported with HLP (20), data in sTBI on antibiotic exposure duration, pulmonary physiology, and secretion drainage remain limited. We therefore assessed the association between HLP and antibiotic exposure in a real-world neurocritical care setting, and we report safety and feasibility to inform future trials.

## Methods

### Study design and participants

This retrospective cohort study was conducted in the intensive care unit of the aforementioned hospital from January 2023 to June 2024. All procedures were strictly in accordance with relevant guidelines and regulations, including the Declaration of Helsinki. The study protocol was approved by the Ethics Committee of the Clinical Medical College of Fujian Medical University (Approval No. K2022-09-061). Data were retrospectively extracted from the clinical information system database of the participating hospital. Given the non-interventional and observational nature of this study and the absence of any physiological risks to patients, the ethics committee waived the requirement for informed consent. All patient data were anonymized and processed in strict compliance with institutional privacy protection protocols.

**Figure 1:**
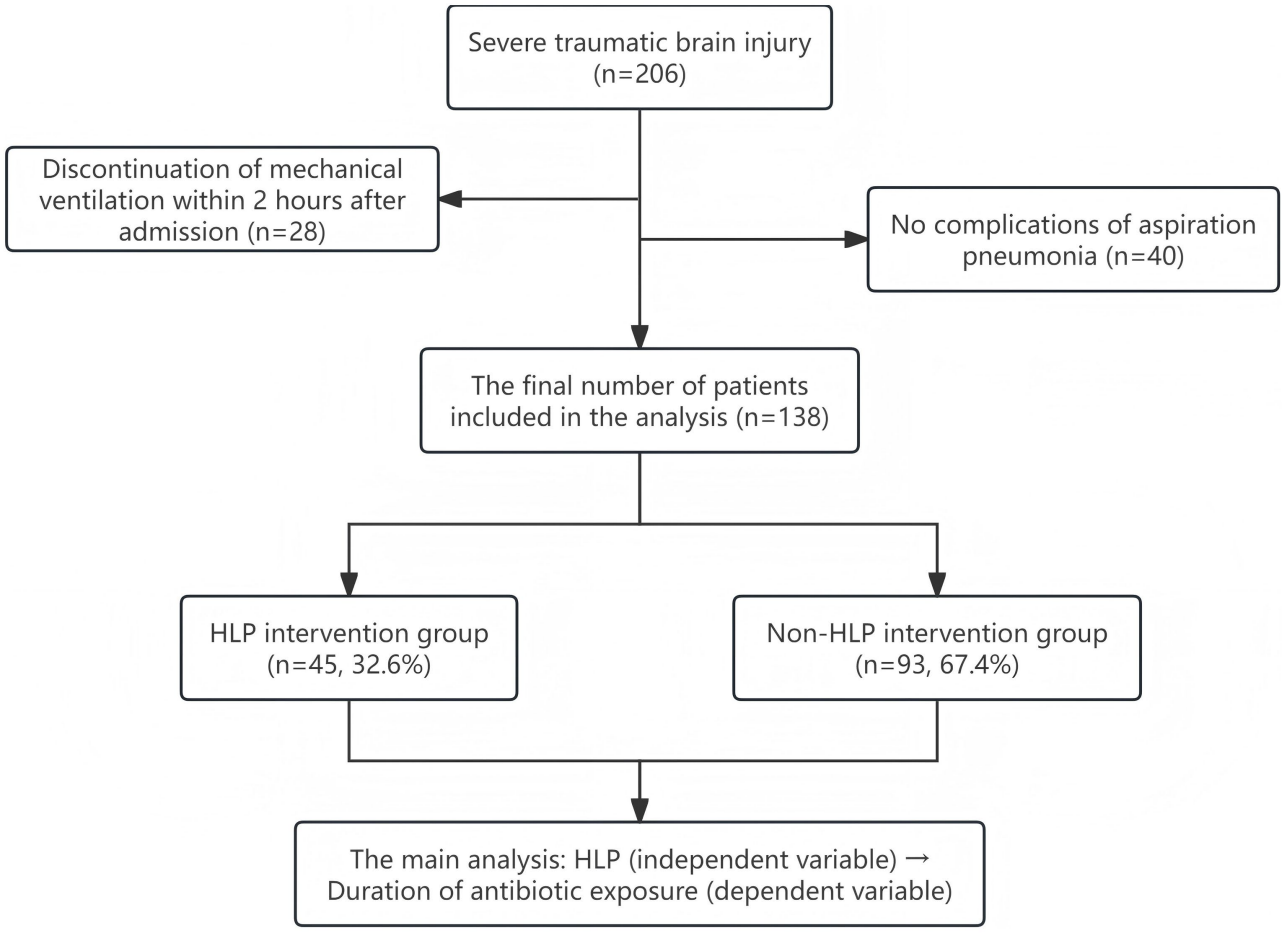
Flowchart of patient selection.

All patients included in this study met the following criteria: (1) Glasgow Coma Scale (GCS) score ≤8; (2) radiologically confirmed aspiration pneumonia; (3) age between 18 and 80 years; (4) ICU stay of at least 7 days; (5) initiation of invasive mechanical ventilation upon admission. Exclusion criteria included: (1) presence of active bleeding, spinal cord injury, or patients in late pregnancy; (2) combined hemothorax and pneumothorax or severe arrhythmias; (3) death or self-discharge within 7 days of admission. Based on the diagnostic criteria for traumatic brain injury (TBI), a total of 863 TBI patients were initially identified. According to the inclusion and exclusion criteria, 206 patients with GCS scores ≤8 who received mechanical ventilation upon admission were selected as severe TBI (sTBI) patients. Among them, 28 patients had mechanical ventilation discontinued within 2 h of admission. Based on chest X-ray or CT findings, 40 patients were diagnosed with simple pneumonia and were excluded from the study. After screening, a total of 138 sTBI patients with aspiration pneumonia were finally included for analysis (see Figure **1**). This study primarily investigated the association between HLP and antibiotic exposure duration, with HLP as the independent variable and antibiotic exposure duration as the dependent variable. The screening results showed that approximately 67.4% (93/138) of the finally selected patients did not receive HLP intervention, while 32.6% (45/138) did. Through a strict screening process, the homogeneity of the patients was ensured, and the impact of confounding factors was minimized to the greatest extent. This provided a solid basis for analyzing the effectiveness of HLP intervention and a certain degree of comparability for subsequent association analysis.

### Intervention

#### Positioning intervention and control

Both groups in this study received standardized evidence-based position care. The control group (non-HLP) implemented head-of-bed (HOB) elevation at 30° (acceptable range 25–35°) and routine position changes every 2 h (15–30° lateral rotation), and routinely performed chest physiotherapy and airway management, with processes in line with guidelines for neurocritical care and pressure ulcer prevention (21).

**Figure 2:**
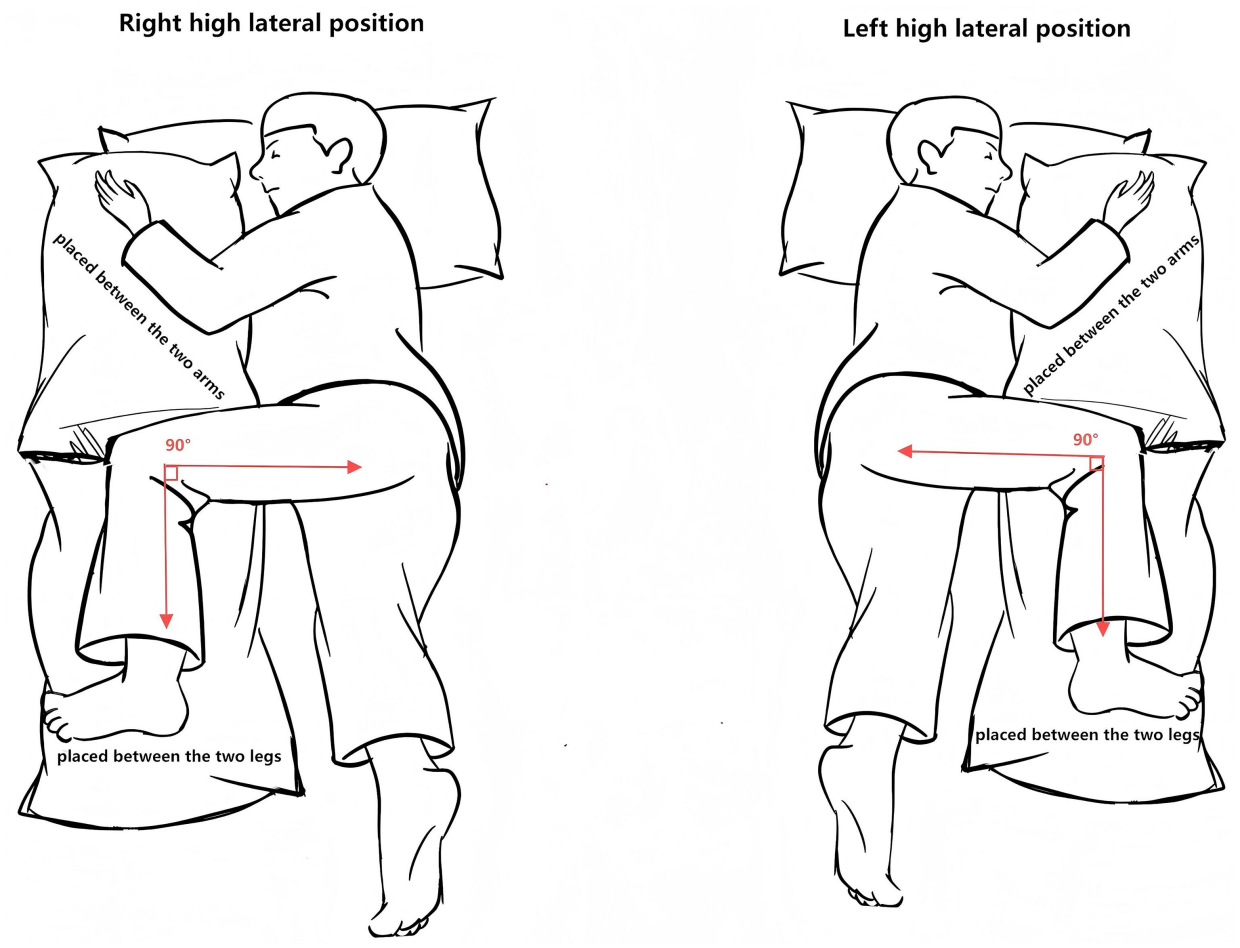
Diagram of the high lateral positioning (HLP) protocol.

The HLP group added high lateral position (HLP) as a positioning intervention on top of the above standard care and implemented it according to a predefined standard operating procedure (SOP). Patients were turned to approximately 90° lateral tilt with 15–30° head-of-bed elevation and a neutral neck position, with all catheters carefully secured. HLP sessions alternated left and right every ~2 h (approximately 2 h on each side), targeting a cumulative daily duration of 10–12 h, based on previous positioning-therapy studies aiming to redistribute ventilation/perfusion and enhance gravitational secretion drainage (20, 22). Throughout HLP, patients were placed on a pressure-relieving mattress to minimize the risk of pressure injury (22). Before initiating HLP, major contraindications (e.g., spinal cord injury, active bleeding, severe arrhythmia) were screened, and catheter patency and skin condition were checked. Vital signs, skin integrity, and respiratory parameters were monitored throughout, and HLP was stopped if ICP exceeded 25 mmHg for ≥ 5 min, mean arterial pressure decreased by > 20%, or persistent hypoxemia or new-onset arrhythmia occurred. Spinal neutrality and line protection were maintained at all times. A detailed step-by-step description of staff roles, turning technique, and device use is provided in Supplementary Appendix 1 and is illustrated in Figure 2.

#### Covariates and pre-intervention physiologic window

To control for the impact of confounding factors on the association between HLP and antibiotic exposure duration, we systematically collected four categories of covariates: (1) baseline characteristics, including sex, age, and comorbidities (hypertension, diabetes, chronic obstructive pulmonary disease [COPD], chronic kidney disease, coronary heart disease); (2) physiological indicators, including Glasgow Coma Scale (GCS) score, APACHE II score, SOFA score, ICP, CPP, MAP, and use of vasopressors; (3) laboratory parameters, including arterial blood gas analysis, inflammatory markers, and related biochemical indicators on the first and seventh days of admission; and (4) sedation depth, assessed using the Richmond Agitation-Sedation Scale (RASS). Based on clinical relevance and prior literature, only a subset of these variables (age, sex, COPD, PaO₂ on day 1, and PaO₂/FiO₂[P/F] ratio on day 7) was entered into the multivariable regression models, whereas the remaining variables were used for descriptive comparisons and sensitivity checks.

In addition, to reduce the selection bias that “more stable patients are more likely to receive HLP,” we systematically collected data before the first implementation of HLP (for the non-HLP group, the corresponding disease course time window was used), including physiological and intensity of care variables: ICP, CPP, MAP, P/F, PaCO₂, ventilator parameters, RASS, exposure to sedation/paralysis, 24-h cumulative fluid balance, and microbiological/antibiotic resistance information. This dataset was used to test the comparability of pre-intervention physiology and care intensity between groups and to inform the selection of clinically relevant covariates for the multivariable models, thereby reducing residual confounding as far as possible in this retrospective design.

#### Outcome definition and antimicrobial stewardship

Primary outcome: antibiotic exposure days, defined at the patient level as the number of calendar days from the first dose of any systemic antibiotic to the final discontinuation of all systemic antibiotics (end − start + 1). Days on which two or more systemic antibiotics were administered concurrently were counted as a single antibiotic exposure day. Antibiotic switches without gaps were considered part of the same treatment episode. An independent Antimicrobial Stewardship Program (ASP) oversaw discontinuation decisions throughout the 18-month period without changes in team or policy. Discontinuation required concurrent, prespecified criteria (23, 24): (a) clinical improvement—temperature < 38 °C for ≥ 24 h; (b) laboratory improvement—procalcitonin ≤ 0.5 ng/mL or ≥ 80% reduction from baseline and normalization of white blood cell count; and (c) radiological improvement—documented by a radiologist on chest X-ray or CT.

All patients received standardized ICU management, including neurosurgical intervention, dehydration to reduce intracranial pressure when indicated, anti-infective treatment, prevention of complications, and enteral nutrition. Sedation/analgesia followed contemporary ICU guidelines (25): propofol, midazolam, or dexmedetomidine were selected based on the condition, and the RASS target was controlled at −3 to −2 during the acute phase of mechanical ventilation; when the condition permitted, a transition to lighter sedation (−2 to 0) was attempted, with assessment every 2 h. Core body temperature was maintained at 36–37 °C using physical cooling methods. Nursing interventions included continuous monitoring of vital signs, airway humidification management, and repositioning and back percussion every 2 h to facilitate sputum expulsion.

### Statistical analysis

Normally distributed variables were presented as mean (SD), skewed variables as median [IQR], and categorical variables as *n* (%). Student’s *t*-test or Mann–Whitney U test was used to compare continuous variables, as appropriate, and Fisher’s exact test was used for categorical variables. Linear regression models (*β*, 95% CI) were used to assess associations between covariates and antibiotic exposure duration. Model assumptions were evaluated using residual plots, which did not reveal major violations.

Three nested linear regression models were specified *a priori*. Model I provided unadjusted estimates of the association between high lateral position (HLP vs. non-HLP) and antibiotic exposure duration without controlling for any covariates. Model II additionally adjusted for demographic variables (age and sex). Model III further incorporated covariates that were judged clinically relevant beforehand and showed at least a modest association with antibiotic exposure in univariable analyses. Multicollinearity among candidate covariates was assessed using variance inflation factors (VIFs). When both arterial PaO₂ and the PaO₂/FiO₂ (P/F) ratio measured on day 7 were entered simultaneously, the two oxygenation indices exhibited pronounced collinearity (VIFs 19.21 and 22.49, respectively), whereas all other covariates had VIFs < 2. Because the P/F ratio better reflects oxygenation under varying FiO₂ conditions, day-7 PaO₂ was removed to avoid redundancy and only the P/F ratio was retained. Consequently, the final Model III included HLP and five covariates—age, sex, COPD, PaO₂ on day 1, and the P/F ratio on day 7—with all VIFs < 2, indicating low collinearity. Statistical significance was set at two-sided *p* ≤ 0.05. Analyses were performed with R 3.3.2 and Free Statistics 1.9.2.

## Results

### Cohort characteristics and clinical outcomes

A total of 138 sTBI patients were finally included in this study. Among them, 82.6% (114/138) were male and 17.4% (24/138) were female, with a mean age of 56.8 ± 16.5 years. The main comorbidities included: epidural hematoma (51.4%), basilar skull fracture (50.7%), subdural hematoma (38.4%), skull fracture (34.1%), and subarachnoid hemorrhage (21.7%). The distribution of intracranial injury combinations is detailed in Supplementary Figure S1. Baseline characteristics did not differ significantly between groups (Table 1), and there was no statistically significant difference in age between the HLP group (*n* = 45) and the non-HLP group (*n* = 93) (59.7 ± 15.5 vs. 55.4 ± 16.8 years, *p* = 0.15). However, some variables showed numerically higher values in the HLP group, so a degree of residual clinical imbalance cannot be excluded.

**Table 1:** Baseline characteristics and outcomes.

Variables	Total (*n* = 138)	sTBI patients		*p*-value
		Non-HLP group n = 93	HLP group n = 45	
Gender				
Male	114 (82.6)	77 (82.8)	37 (82.2)	0.934
Female	24 (17.4)	16 (17.2)	8 (17.8)	
Age, years	56.8 ± 16.5	55.4 ± 16.8	59.7 ± 15.5	0.151
Epidural hematoma, *n* (%)	71 (51.4)	48 (51.6)	23 (51.1)	0.956
Subdural hematoma, *n* (%)	53 (38.4)	37 (39.8)	16 (35.6)	0.632
Basal fracture, *n* (%)	70 (50.7)	48 (51.6)	22 (48.9)	0.764
Subarachnoid hemorrhage, *n* (%)	30 (21.7)	21 (22.6)	9 (20)	0.730
Calvarial fractures, *n* (%)	47 (34.1)	30 (32.3)	17 (37.8)	0.521
Diabetes mellitus, *n* (%)	27 (19.6)	20 (21.5)	7 (15.6)	0.409
IHD, *n* (%)	4 (2.9)	2 (2.2)	2 (4.4)	0.596
Hypertension, *n* (%)	52 (37.7)	32 (34.4)	20 (44.4)	0.254
COPD, *n* (%)	7 (5.1)	6 (6.5)	1 (2.2)	0.427
CHD, *n* (%)	7 (5.1)	5 (5.4)	2 (4.4)	1.000
MAP (mmHg)	86.2 ± 16.9	86.8 ± 17.3	84.8 ± 16.0	0.501
ICP (mmHg)	11 (9, 14)	11 (9, 14)	11 (8, 14)	0.943
CPP (mmHg)	76 (64, 86)	78 (65, 86)	72 (64, 86)	0.516
Vasoactive drugs, *n* (%)	62 (44.9)	41 (44.1)	21 (46.7)	0.775
GCS score	5.0 ± 1.9	5.2 ± 2.0	4.7 ± 1.6	0.167
APACHE II, score	30.7 ± 7.9	30.4 ± 8.5	31.3 ± 6.4	0.549
SOFA, score	12.0 ± 3.4	11.8 ± 3.8	12.4 ± 2.2	0.331
RASS, score	−3.6 ± 0.7	−3.5 ± 0.8	−3.7 ± 0.5	0.088
pH day 1	7.4 ± 0.1	7.4 ± 0.1	7.4 ± 0.1	0.895
PaCO₂ day 1	42.9 ± 7.6	43.3 ± 8.0	42.0 ± 6.7	0.336
PaO₂ day 1	131.2 ± 47.1	132.9 ± 50.2	127.6 ± 40.0	0.536
P/F day 1	286.1 ± 118.1	292.5 ± 125.5	273.0 ± 101.3	0.364
PCT day 1	1.2 (0.7, 3.0)	1.3 (0.8, 3.0)	1.0 (0.5, 2.5)	0.171
WBC day 1	14.0 ± 4.2	13.7 ± 3.3	14.5 ± 5.6	0.261
pH day 7	7.4 ± 0.1	7.4 ± 0.1	7.4 ± 0.1	0.945
PaCO₂ day 7	41.9 ± 8.4	43.1 ± 9.7	39.5 ± 3.9	0.018
PaO₂ day 7	157.6 ± 49.3	153.5 ± 49.9	166.2 ± 47.5	0.158
P/F day 7	349.3 ± 103.6	342.0 ± 106.8	364.4 ± 96.2	0.235
PCT day 7	0.4 (0.2, 1.2)	0.5 (0.2, 1.3)	0.2 (0.1, 0.5)	0.010
WBC day 7	11.3 ± 3.8	12.0 ± 3.8	10.0 ± 3.5	0.004
28-day mortality, *n* (%)	21 (15.2)	17 (18.3)	4 (8.9)	0.150
Mechanical ventilation, days	8.0 (6.0, 10.8)	9.0 (7.0, 11.0)	7.0 (5.0, 8.0)	0.004
Length of ICU stay	12.1 ± 5.7	12.6 ± 6.1	11.0 ± 4.8	0.135
Duration of antibiotic exposure	11.5 ± 5.6	12.4 ± 6.1	9.5 ± 4.0	0.004
Tracheostomy, *n* (%)	22 (15.9)	19 (20.4)	3 (6.7)	0.038

IHD: Ischemic Heart Disease; COPD: chronic obstructive pulmonary disease; CHD: coronary heart disease; MAP: mean arterial pressure; ICP: intracranial pressure; CPP: cerebral perfusion pressure; GCS: Glasgow coma scale; APACHE II: Acute Physiology and Chronic Health Evaluation II score; SOFA: Sequential Organ Failure Assessment; RASS: Richmond Agitation-Sedation Scale; PaCO_2_: Partial pressure of carbon dioxide in arterial blood; PaO_2_: Partial pressure of oxygen in arterial blood; P/F: PaO_2_/FiO_2_; PCT: procalcitonin; WBC: white blood cell count; ICU: intensive care unit.

**Figure 3:**
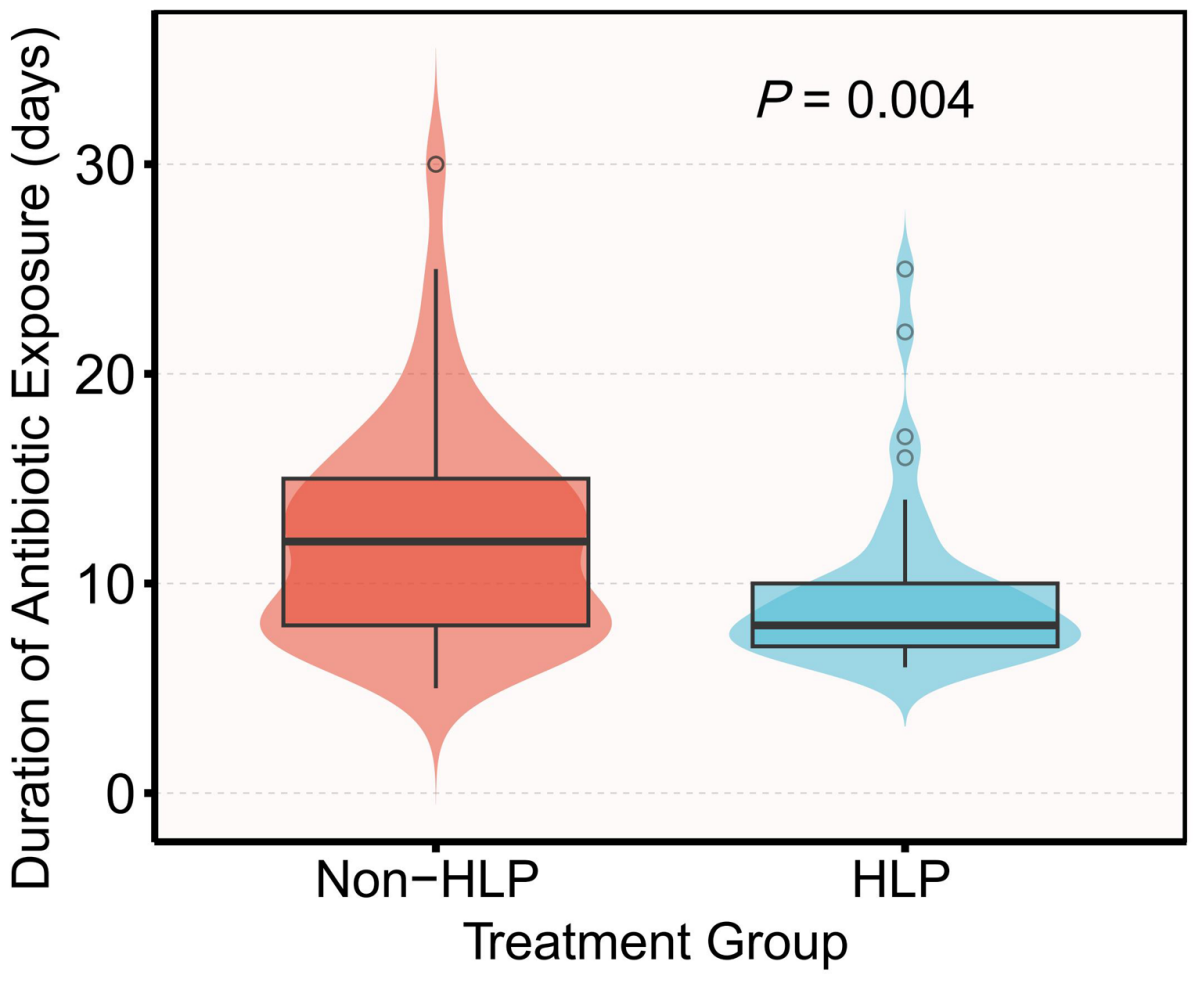
Violin and box plots of antibiotic exposure duration by group (HLP vs. non-HLP). Mean antibiotic exposure was 12.4 ± 6.1 days in the non-HLP group and 9.5 ± 4.0 days in the HLP group (mean difference −2.9 days; *p* = 0.004 by Mann–Whitney *U* test).

Pre-intervention physiological profiles did not show statistically significant differences between groups (Table 1). Median ICP was 11 mmHg in both groups (IQR 8–14 vs. 9–14; *p* = 0.94), and CPP medians were 72 (64–86) vs. 78 (65–86) mmHg (*p* = 0.52); other oxygenation, ventilation, and circulatory indices showed no material imbalances. On day 7, the HLP group had significantly lower PaCO₂, procalcitonin (PCT), and white blood cell (WBC) levels, fewer days of mechanical ventilation, a shorter duration of antibiotic exposure, and a lower tracheostomy rate compared with the non-HLP group (all *p* < 0.05; Table 1 and Figure 3).

Regarding safety, 2 cases (4.4%) in the HLP group were prematurely terminated due to triggering the predefined stopping criteria: 1 case with ICP > 25 mmHg for ≥5 min and 1 case with MAP decrease >20% combined with hypoxia. In the non-HLP group, 4 cases (4.3%) experienced similar events (2 cases with elevated ICP, 1 case with MAP decrease, and 1 case with new-onset arrhythmia). There was no statistically significant difference in event occurrence rates between the two groups (Fisher’s exact test, OR = 1.03, 95% CI 0.18–5.87, *p* = 1.000). Given the very low event counts, confidence intervals around these estimates are wide, and the analysis is underpowered to rule out small to moderate differences; therefore, the safety results should be viewed as descriptive (Supplementary Tables S2, S3).

### Factors associated with antibiotic exposure

We then examined factors associated with the duration of antibiotic exposure using univariable and multivariable linear regression models. The results of univariate analysis showed that ICP and CPP before intervention were not significantly linearly correlated with the duration of antibiotic exposure (both *p* > 0.80). Protective factors included HLP (*β* = −2.93; 95% CI, −4.90 to −0.96; *p* = 0.004), PaO₂ on day 1 (β = −0.02; 95% CI, −0.04 to 0; *p* = 0.039), PaO₂ on day 7 (*β* = −0.02; 95% CI, −0.04 to 0; *p* = 0.018), and P/F ratio on day 7 (*β* = −0.01; 95% CI, −0.02 to 0; *p* = 0.011), indicating that better oxygenation was associated with shorter antibiotic exposure. Conversely, COPD (*β* = 9.72; 95% CI, 5.71 to 13.74; *p* < 0.001) and days of mechanical ventilation (β = 1.03; 95% CI, 0.92 to 1.14; *p* < 0.001) were associated with longer antibiotic exposure (Table 2).

**Table 2:** Univariate analysis for duration of antibiotic exposure.

Covariate	*β* (95%CI)	*P*-value
High lateral position	−2.93 (−4.9,-0.96)	0.004
Gender	−0.07 (−2.58,2.45)	0.957
Age, per 1 year	0.04 (−0.01,0.10)	0.128
Epidural hematoma	1.67 (−0.22,3.55)	0.082
Subdural hematoma	0.99 (−0.96,2.94)	0.318
Basal fracture	1.04 (−0.85,2.94)	0.278
Subarachnoid hemorrhage	0.4 (−1.91,2.71)	0.731
Calvarial fractures	0.07 (−1.94,2.08)	0.945
Diabetes mellitus	1.56 (−0.83,3.95)	0.198
IHD	0.79 (−4.89,6.46)	0.784
Hypertension	1.07 (−0.89,3.03)	0.281
COPD	9.72 (5.71,13.74)	< 0.001
CHD	1.44 (−2.89,5.78)	0.511
MAP	0 (−0.05,0.06)	0.944
ICP	0.03 (−0.26, 0.28)	0.834
CPP	0 (−0.05,0.06)	0.981
Vasoactive drugs	0.85 (−1.06,2.76)	0.382
GCS score	0.1 (−0.4,0.61)	0.685
APACHE II score	−0.07 (−0.19,0.05)	0.265
SOFA score	−0.03 (−0.32,0.25)	0.819
RASS score	0.58 (−0.78,1.94)	0.398
PH day 1	0.02 (−1.04,1.07)	0.975
PaCO_2_ day 1	−0.01 (−0.13,0.12)	0.913
PaO₂ day 1	−0.02 (−0.04,0.00)	0.039
P/F day 1	−0.01 (−0.01,0.00)	0.199
PCT day 1	0.04 (−0.06,0.14)	0.395
WBC day 1	−0.15 (−0.38,0.07)	0.179
PH day 7	0.98 (−0.12,2.09)	0.080
PaCO_2_ day 7	−0.04 (−0.15,0.07)	0.483
PaO_2_ day 7	−0.02 (−0.04,0.00)	0.018
P/F day 7	−0.01 (−0.02, 0.00)	0.011
PCT day 7	−0.03 (−0.13,0.08)	0.579
WBC day 7	−0.11 (−0.36,0.14)	0.396

Continuous covariates are modeled per unit increase unless otherwise specified. β coefficients represent the change in antibiotic exposure days per unit increase in the covariate; 95% CIs are shown in parentheses. p values are two-sided. Abbreviation: IHD: Ischemic Heart Disease; COPD: chronic obstructive pulmonary disease; CHD: coronary heart disease; MAP: mean arterial pressure; ICP: Intracranial Pressure; CPP: Cerebral Perfusion Pressure; GCS: Glasgow coma scale; APACHE II: Acute Physiology and Chronic Health Evaluation II score; SOFA: Sequential Organ Failure Assessment; RASS: Richmond Agitation-Sedation Scale; PaCO_2_: Partial pressure of carbon dioxide in artery blood; PaO_2_: Partial pressure of oxygen in artery blood; P/F: PaO_2_/FiO_2_; PCT: procalcitonin; WBC: white blood count; ICU: intensive care unit.

### Multivariable models

Model I (without adjusting for any covariates) showed that HLP (*β* = −2.93; 95% CI -4.9 to −0.96; *p* = 0.004), PaO₂ on day 1 (*β* = −0.21; 95% CI -0.41 to −0.01; *p* = 0.039), and P/F ratio on day 7 (*β* = −0.12; 95% CI -0.21 to −0.03; *p* = 0.011) were each associated with shorter antibiotic exposure in univariable analysis. COPD (*β* = 9.72; 95% CI 5.71 to 13.74; *p* < 0.001) was a significant risk factor.

Model II (adjusted for age and gender): HLP (*β* = −3.17; 95% CI, −5.13 to −1.22; *p* = 0.002) and P/F ratio on day 7 (*β* = −0.11; 95% CI, −0.2 to −0.01; *p* = 0.027) remained protective factors. COPD (*β* = 9.69; 95% CI, 5.43 to 13.94; *p* < 0.001) remained a significant risk factor.

Model III (further adjusted for age, gender, chronic obstructive pulmonary disease, PaO₂ value on day 1, and P/*F* value on day 7): HLP (*β* = −2.58; 95% CI –4.44 to −0.71; *p* = 0.008) remained a protective factor for antibiotic exposure duration in patients with severe traumatic brain injury. Chronic obstructive pulmonary disease (*β* = 8.78; 95% CI, 4.42 to 13.13; *p* < 0.001) remained a significant risk factor for antibiotic exposure duration in patients with severe traumatic brain injury (see Table **3**).

**Table 3:** Multivariate analyses of risk factors associated with duration of antibiotic exposure.

	Model I		Model II		Model III	
	*β* (95%CI)	*P-*value	*β* (95%CI)	*P-*value	*β* (95% CI)	*P-*value
Age						
Per 10	0.45 (−0.13,1.02)	0.128	0.45 (−0.13,1.03)	0.128	0.45 (−0.13,1.03)	0.128
<65	Reference		Reference		Reference	
≥65	2.26 (0.29,4.24)	0.025	2.75 (−0.44,5.95)	0.094	2.75 (−0.44,5.95)	0.094
Gender						
Male	−0.07 (−2.58,2.45)	0.957				
Female	Reference		Reference		Reference	
HLP						
No	Reference		Reference		Reference	
Yes	−2.93 (−4.9,–0.96)	0.004	−3.17 (−5.13,–1.22)	0.002	−2.58 (−4.44,–0.71)	0.008
COPD						
No	Reference		Reference		Reference	
Yes	9.72 (5.71,13.74)	<0.001	9.69 (5.43,13.94)	<0.001	8.78 (4.42,13.13)	<0.001
PaO₂ day 1 (per 10)	−0.21 (−0.41,–0.01)	0.039	−0.18 (−0.39,0.04)	0.115	−0.09 (−0.33,0.14)	0.445
P/F Day 7 (per 10)	−0.12 (−0.21,–0.03)	0.011	−0.11 (−0.2,–0.01)	0.027	−0.09 (−0.19,0.01)	0.084

Data presented are β and 95% CIs. β (days) with 95% CI from linear regression; two-sided *P-*values. Model I: Did not adjust any covariates. Model II: Adjusted age, gender. Model III: Adjusted age, gender, COPD, PaO_2_ Day 1, P/F Day 7. Abbreviation: HLP: High Lateral Position; COPD: Chronic Obstructive Pulmonary Disease; PaO_2_: Partial pressure of oxygen in artery blood; P/F: PaO_2_/FiO_2_; Per 10: denotes the linear effect on the outcome (days) for every 10-unit increase in the independent variable.

## Discussion

This single-center retrospective cohort observed an association between high lateral positioning (HLP) and a shorter duration of antimicrobial exposure, along with trends toward fewer days of mechanical ventilation and a lower tracheostomy rate. These endpoints were not prespecified and were examined as exploratory post-hoc analyses; the study was not powered for them and no adjustment for multiple comparisons was performed, so these observations should be interpreted with caution.

From a physiological perspective, combining a modest head-up tilt (approximately 15–30°) with a 90° lateral position may better preserve relative stability of ICP/CPP through favorable extracranial venous return and intrathoracic pressure gradients. Concurrently, gravity may facilitate secretion drainage and improve ventilation–perfusion matching, thereby accelerating infection control and shortening the course of antimicrobial therapy. If HLP is indeed associated with reduced antimicrobial exposure, direct drug costs and related supportive-care expenditures could theoretically be reduced, consistent with prior estimates of the economic burden associated with antibiotic resistance and prolonged antibiotic treatment (26). However, such economic effects are highly context-dependent across institutions and pricing structures, and no formal economic evaluation was performed in this study. Prospective work using micro-costing and cost-effectiveness or cost-utility analyses will be needed to quantify and verify these signals.

Under the retrospective design framework, we attempted to mitigate the selection bias that “more stable patients are more likely to receive HLP” by systematically collecting immediate data before the first HLP session, with corresponding data extracted at the same disease-course node in the non-HLP group. There were no systematic differences in ICP, CPP, sedation depth, intensity of care, fluid balance, or microbial resistance patterns between groups (all *p* > 0.05), suggesting broadly comparable pre-intervention physiological profiles. Nonetheless, these strategies can only partially address confounding, and unmeasured factors such as subtle differences in disease trajectory or team preferences may still have influenced HLP assignment. As a result, the observed associations should be interpreted cautiously as hypothesis-generating and require confirmation in randomized controlled trials.

In terms of safety and feasibility, the premature termination rate in the HLP group was 4.4%, with a median daily cumulative implementation of 10.0 (10.0–12.0) h and a target achievement rate of 95.6%. The non-HLP group also recorded 4 cases (4.3%) of similar events, with no statistically significant difference between the two groups (*p* = 1.000). No adverse events such as catheter dislodgement or pressure ulcers occurred throughout the study. The above data suggest that under high-level monitoring and standardized procedures, HLP is well-tolerated in the short term, with risk signals no higher than those of routine position management.

Although the 28-day mortality rate in the HLP group was numerically lower than that in the non-HLP group (8.9% vs. 18.3%), the difference did not reach statistical significance (*p* = 0.150). In sTBI, death is more often dominated by the severity of neurotrauma, intracranial lesions, and secondary brain injury (27). Systematic evidence in the field of critical care indicates that improving pulmonary intermediate endpoints such as oxygenation alone does not necessarily translate into mortality benefits, reinforcing the uncertainty of translating intermediate endpoints into hard outcomes (28). This evidence is consistent with the phenomenon observed in our study, where pulmonary indicators improved but short-term mortality did not significantly decrease. Therefore, we cautiously interpret the mortality results as hypothesis-generating: The potential benefits of HLP are more likely to be reflected in the control of complications and resource utilization rather than short-term survival.

In sTBI, depression of the respiratory drive, sedation, and mechanical ventilation can impair mucociliary clearance and cough reflex, leading to secretion retention and a vicious cycle of “infection–inflammation–reinfection,” thereby prolonging ICU length of stay (29). Under gravitational influence, HLP may promote secretions from upper-lobe bronchi to drain into larger airways and improve ventilation–perfusion matching, reducing the risks of mucus accumulation, atelectasis, and infection; the observed decline in PaCO₂ on day 7 (*p* < 0.05) in this study is consistent with this direction of effect (30–32). In addition, a high lateral position can expand chest-wall excursion and optimize diaphragmatic–abdominal muscle synergy, increasing peak expiratory flow and facilitating sputum clearance (33). These mechanistic clues provide a physiologically plausible backdrop for the directional associations observed here.

We also found that concomitant COPD was consistently associated with longer antibiotic exposure (*β* = 8.78, *p* < 0.001). This is biologically plausible, as COPD is characterized by chronic airway inflammation, airflow limitation, and structural changes such as small airway disease and emphysema, which are strongly linked to future exacerbations and recurrent lower respiratory events (34, 35). These exacerbations are frequently infection-driven and commonly managed with systemic antibiotics, so patients with COPD tend to experience a higher cumulative burden of antibiotic-treated episodes over time, particularly in high-risk phenotypes with structural disease (34, 35). In our cohort, HLP was associated with fewer days of mechanical ventilation and reductions in inflammatory markers (PCT ↓ 0.3 ng/mL, WBC ↓ 2.0 × 10 (9)/L), suggesting a potential role as a non-pharmacological adjunct to short-course antibiotic strategies, in line with evidence supporting shorter antibiotic courses in appropriately selected critically ill patients (36). However, these observations remain hypothesis-generating and require validation through randomized trials and stratified subgroup analyses in patients with COPD.

In summary, HLP was consistently associated with a shorter duration of antimicrobial exposure in this study and showed several favorable directional signals. Given design limitations, multicenter randomized controlled trials are warranted, incorporating standardized and quantified comparator SOPs, structured safety endpoints, and standardized antibiotic discontinuation pathways, while evaluating both intermediate pulmonary outcomes and patient-centered hard endpoints to test the effectiveness, safety, and generalizability of HLP.

## Conclusion

In this retrospective cohort, HLP was associated with a shorter duration of antibiotic exposure and showed trends toward fewer days of mechanical ventilation and a lower tracheostomy rate. Given the nonrandomized, single-center design and potential residual confounding, these findings are exploratory and should be confirmed in prospective randomized studies. Future randomized controlled trials should incorporate standardized ventilation and positioning protocols, predefined safety stopping criteria, and quantitative measures of secretion clearance and pulmonary mechanics to more definitively evaluate the causal effects of HLP on antibiotic exposure and clinically important outcomes.

## Data Availability

The original contributions presented in the study are included in the article/Supplementary material, further inquiries can be directed to the corresponding author/s.

## Glossary

HLP: High Lateral Position
COPD: Chronic Obstructive Pulmonary Disease
sTBI: Severe Traumatic Brain Injury
TBI: Traumatic Brain Injury
PP: prone positioning
APACHE II: Acute Physiology and Chronic Health Evaluation II score
GCS: Glasgow coma scale
ICU: intensive care unit
PCT: procalcitonin
SOFA: Sequential Organ Failure Assessment
MAP: mean arterial pressure
ICP: Intracranial Pressure
CPP: Cerebral Perfusion Pressure
RASS: Richmond Agitation-Sedation Scale
P/F: PaO2/FiO2
WBC: White Blood Cell Count
DM: diabetes mellitus
CKD: chronic kidney disease
CHD: coronary heart disease
CI: confidence intervals
IQR: interquartile range
PEF: Peak Expiratory Flow
SOP: Standard Operating Procedure
ASP: Antimicrobial Stewardship Program
