# Supplementary material for: The effect of high lateral position on antibiotic exposure duration in patients with severe traumatic brain injury: a retrospective observational cohort study
Source: Front Med (Lausanne). 2026 Jan 5;12:1665953. doi: 10.3389/fmed.2025.1665953 (PMC12813169; doi:10.3389/fmed.2025.1665953)
Supplement: Supplementary file 1 [file Table_1.docx]

**Supplementary Material for “The Effect of High Lateral Position on Antibiotic Exposure Duration in Patients with Severe Traumatic Brain Injury: A Retrospective Observational Cohort Study”**

**Supplementary Appendix 1. Standard operating procedure for high lateral positioning (HLP)**

1. Purpose and scope

This standard operating procedure (SOP) describes the implementation of high lateral positioning (HLP) in adult patients with severe traumatic brain injury (sTBI) receiving mechanical ventilation in the neurocritical care unit. The aim is to optimize ventilation/perfusion distribution and secretion drainage while preserving intracranial pressure (ICP) and cerebral perfusion pressure (CPP) stability.

2. Indications and contraindications

2.1 Indications

- Adult patients with sTBI requiring invasive mechanical ventilation;

- Presence of, or high risk for, aspiration pneumonia or other lower respiratory tract infection;

- Hemodynamic status that is stable or can be stabilized with vasoactive support;

- No immediate contraindication to lateral tilt based on the attending physician’s judgment.

2.2 Contraindications (screened before each HLP session)

- Known or suspected spinal cord injury for which lateral tilt is unsafe;

- Active or uncontrolled bleeding (e.g., intracranial, thoracic, or abdominal);

- Hemodynamic instability not responsive to resuscitation (e.g., persistent MAP < 65 mmHg despite vasoactive support);

- Severe arrhythmia at baseline or during position changes;

- Any other condition judged by the treating team to render HLP unsafe at that time.

3. Preparation

- Verify patient eligibility and confirm absence of the contraindications listed above.

- Confirm patency and secure fixation of all invasive lines and tubes, including:

- Endotracheal or tracheostomy tube;

- Central venous and arterial catheters;

- External ventricular drain and ICP monitoring lines;

- Urinary catheter and feeding tubes.

- Ensure that the patient lies on a pressure-relieving mattress to reduce the risk of pressure injury.

- Adjust analgesia and sedation as needed to maintain comfort and prevent agitation during turning, in line with unit protocols.

- Prepare positioning aids:

- An angle-measuring device (e.g., goniometer or digital inclinometer);

- Pillows or foam wedges to support the trunk and limbs;

- Protective padding for bony prominences (e.g., elbows, knees, ankles).

4. Positioning procedure

- HLP is performed by two trained staff members working together.

- Starting from the supine position, use the angle-measuring device to rotate the patient to a lateral tilt of 90° ± 5° while maintaining:

- Head-of-bed elevation at 15–30°;

- A neutral neck position without excessive flexion, extension, or rotation.

- After turning, re-check the position of all catheters and lines to ensure that:

- The endotracheal or tracheostomy tube is midline without undue tension;

- Central venous and arterial lines are not kinked, compressed, or pulled;

- The external ventricular drain and ICP transducer (if present) remain at the correct reference level;

- No tubing is compressed between the patient and the bed or bed rails.

- Stabilize the patient in the lateral position using pillows or wedges under the trunk, back, and extremities as needed, avoiding direct pressure on the abdomen and chest.

- Maintain HLP on one side for approximately 2 hours, then carefully turn the patient to the opposite side using the same procedure. A typical cycle is:

- “~2 hours on the left side → ~2 hours on the right side”.

- Aim for a cumulative daily HLP duration of 10–12 hours, distributed over multiple sessions as tolerated.

5. Monitoring and safety

- Continuously monitor:

- ICP and CPP ;

- Mean arterial pressure (MAP) and heart rate;

- Oxygen saturation (SpO₂) and other respiratory parameters;

- Skin integrity at pressure points and the condition of all catheter insertion sites.

- Prespecified stopping criteria for HLP:

- ICP > 25 mmHg for ≥ 5 minutes despite standard interventions;

- MAP decrease > 20% from baseline that is not rapidly reversible with routine measures;

- Persistent hypoxemia (e.g., SpO₂ < 90% or PaO₂ < 60 mmHg) not corrected by ventilator adjustment;

- New-onset clinically significant arrhythmia.

- If any stopping criterion is met:

- Return the patient to a safer position (usually 30° head-of-bed semi-recumbent);

- Evaluate and treat the underlying cause (e.g., adjust sedation, fluids, vasopressors);

- Document the event, the side of HLP, the timing, and the clinical response.

6. Line protection and spinal neutrality

- Throughout HLP sessions, maintain spinal neutrality with appropriate support under the head, shoulders, pelvis, and legs.

- Avoid twisting of the torso relative to the pelvis and prevent excessive neck rotation.

- Reassess catheter and line security after any repositioning or bed or head-of-bed adjustment.

- The overall HLP protocol and patient posture are illustrated in the main manuscript (Figure 2).

**Supplementary Figure S1: UpSet plot of intracranial injury combinations.**

**
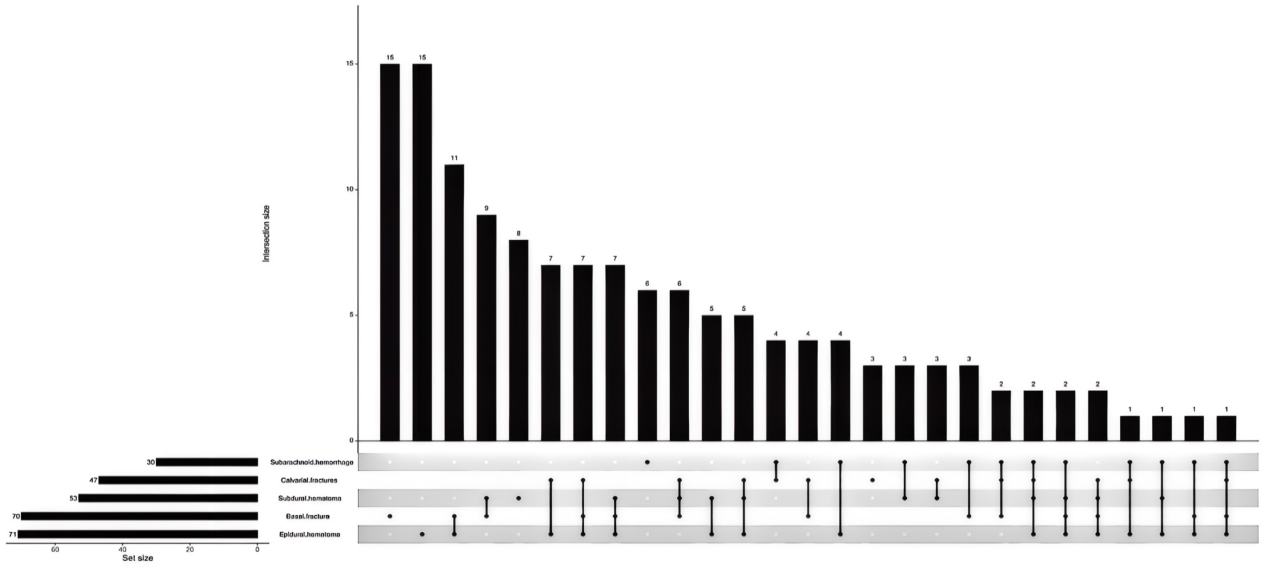
**

**Supplementary Table S1. Additional clinical variables by group (HLP vs Non-HLP)**

| **Variable** | **HLP Group**  **(n = 45)** | **Non-HLP Group (n = 93)** | **P value** |
| --- | --- | --- | --- |
| RASS Day 7 | -3.00 [-3.00–-2.00] | -3.00 [-4.00–-2.00] | 0.119 |
| Cumulative fluid balance Day 7 (mL) | 2165.00 [1528.00–3285.00] | 1999.00 [1472.00–2757.00] | 0.298 |
| Propofol use, n (%) | 27 (60.0%) | 57 (61.3%) | 1.000 |
| Midazolam use, n (%) | 20 (44.4%) | 47 (50.5%) | 0.624 |
| Dexmedetomidine use, n (%) | 17 (37.8%) | 37 (39.8%) | 0.968 |
| Neuromuscular blockade use, n (%) | 4 (8.9%) | 5 (5.4%) | 0.473 |
| Klebsiella pneumoniae, n (%) | 12 (26.7%) | 25 (26.9%) | 1.000 |
| Acinetobacter baumannii, n (%) | 9 (20.0%) | 18 (19.4%) | 1.000 |
| Pseudomonas aeruginosa, n (%) | 8 (17.8%) | 15 (16.1%) | 1.000 |
| Staphylococcus aureus, n (%) | 2 (4.4%) | 3 (3.2%) | 0.661 |
| Other, n (%) | 14 (31.1%) | 32 (34.4%) | 0.847 |
| ESBL positive, n (%) | 2 (4.4%) | 3 (3.2%) | 0.661 |
| Carbapenem-resistant Gram-negative (CR-GN), n (%) | 1 (2.2%) | 2 (2.2%) | 1.000 |
| Multidrug-resistant organism (any), n (%) | 2 (4.4%) | 2 (2.2%) | 0.596 |

Notes: Continuous/ordinal variables are presented as median [IQR] and compared using the Mann–Whitney U test. Categorical variables are n (%) and compared using χ² or Fisher’s exact test, as appropriate. Fluid balance denotes cumulative balance at Day 7. Abbreviations: ESBL, extended-spectrum β-lactamase; CR-GN, carbapenem-resistant Gram-negative; MDR, multidrug-resistant; NMB, neuromuscular blockade; RASS, Richmond Agitation–Sedation Scale.

**Supplementary Table S2. Feasibility of High Lateral Positioning (HLP) — Dose and Adherence (HLP group only)**

| Indicator | Value |
| --- | --- |
| Planned daily target for HLP | 10–12 h/day |
| Actual HLP duration, mean±SD | 10.5 ± 1.1 h/day |
| Daily cumulative HLP duration, median [IQR] | 10.0 (10.0–12.0) h/day |
| Achievement rate (≥10 h/day), n/N (%) | 43/45 (95.6 %) |

Notes . The HLP regimen was planned as a “dose-defined” protocol of 10–12 h/day with side alternation approximately every 2 hours. “Achievement” was defined a priori as ≥10 h/day, quantified from nursing logs (hour-by-hour positioning records).

**Supplementary Table S3. Safety events meeting prespecified stopping criteria**

| **Variables** | **Total (n = 138)** | **sTBI Patients** | | **OR (95% CI)** | **P value** |
| --- | --- | --- | --- | --- | --- |
|  |  | Non-HLP Group n=93 | HLP Group n=45 |  |  |
| Any event meeting stopping criteria, n (%) | 6 (4.3) | 4 (4.3) | 2 (4.4) | 1.03 (0.18–5.87) | 1.000 |
| ICP >25 mmHg ≥5 min, n (%) | 3 (2.2) | 2 (2.2) | 1 (2.2) | — | — |
| MAP drop >20% with hypoxemia, n (%) | 2 (1.4) | 1 (1.1) | 1 (2.2) | — | — |
| New arrhythmia, n (%) | 1 (0.7) | 1 (1.1) | 0 (0.0) | — | — |

Statistical note: The composite row (“Any event…”) was compared using Fisher’s exact test (two-sided): OR = 1.03, 95% CI 0.18–5.87, P = 1.000. Subcategory rows were not tested due to sparse counts and unstable estimates. Non-HLP events are background clinical events recorded under the same stopping-criteria definitions and are not interpreted as “tolerance failures”.
